# Azasteroid Alkylators as Dual Inhibitors of AKT and ERK Signaling for the Treatment of Ovarian Carcinoma

**DOI:** 10.3390/cancers12051263

**Published:** 2020-05-16

**Authors:** Panagiotis Dalezis, Eleni Geromichalou, Aikaterini Polonifi, Sofia Sagredou, Nikolaos Nikoleousakos, Michael Nikolaou, Vasiliki Sarli, Mihalis I. Panayiotidis, Dimitrios T. Trafalis

**Affiliations:** 1 Laboratory of Pharmacology, Medical School, National & Kapodistrian University of Athens, 11527 Athens, Greece; pdalezis@med.uoa.gr (P.D.); elena_geromich@outlook.com (E.G.); apolonif@med.uoa.gr (A.P.); sofiasagr@hotmail.com (S.S.); nikosakos@hotmail.com (N.N.); nikolaoumike@hotmail.com (M.N.); 2 Department of Chemistry, Aristotle University of Thessaloniki, 54124 Thessaloniki, Greece; sarli@chem.auth.gr; 3 Department of Electron Microscopy & Molecular Pathology, The Cyprus Institute of Neurology & Genetics, 2371 Nicosia, Cyprus; 4 The Cyprus School of Molecular Medicine, The Cyprus Institute of Neurology & Genetics, 1683 Nicosia, Cyprus

**Keywords:** lactam steroidal alkylating esters, aniline-mustard alkylators, melphalan, ovarian carcinoma, xenografts, ERK, AKT

## Abstract

(1) Background: Previous findings show that lactam steroidal alkylating esters display improved therapeutic efficacy with reduced toxicity. The aim of this study was to evaluate the anticancer activity of two newly synthesized aza-steroid alkylators (ENGA-L06E and ENGA-L08E) against human ovarian carcinoma cells, and consequently, the dual inhibition of RAS/PI3K/AKT and RAS/RAF/MEK/ERK signaling pathways, both of which are closely associated with ovarian cancer; (2) Methods: The in vitro cytostatic and cytotoxic effects of ENGA-L06E and ENGA-L08E were evaluated in a panel of five human ovarian cancer cell lines, as well as in in vivo studies. ENGA-L06E and ENGA-L08E, in addition to another two aniline-mustard alkylators, POPAM and melphalan (L-PAM), were utilized in order to determine the acute toxicity and antitumor efficacy on two human ovarian xenograft models. Also, in silico studies were performed in order to investigate the dual inhibition of ENGA-L06E and ENGA-L08E on RAS/PI3K/AKT and RAS/RAF/MEK/ERK signaling pathways; (3) Results: Both, in vitro and in vivo studies demonstrated that ENGA-L06E and ENGA-L08E were significantly more effective with a lower toxicity profile in comparison to POPAM and L-PAM alkylators. Moreover, in silico studies demonstrated that the two new aza-steroid alkylators could act as efficient inhibitors of the phosphorylation of AKT and ERK1/2 molecules; and (4) Conclusions: Both ENGA-L06E and ENGA-L08E demonstrated high anticancer activity through the inhibition of the PI3K-AKT and KRAS-ERK signaling pathways against human ovarian carcinoma, and thus constituting strong evidence towards further clinical development.

## 1. Introduction

Ovarian carcinoma (OC) is the most lethal gynecological malignancy due to drug resistance in current therapeutic approaches. There are several histological types of OC that are characterized by distinct molecular genetic alterations associated with different histopathological features. On the other hand, the understanding of the culpable molecular pathways is necessary for the development of novel therapeutics. Nowadays, the therapeutic strategy depends more on tumor stage and grade than on the histological type. The World Health Organization (WHO) classifies ovarian neoplasms according to their histological differentiation, namely epithelial, sex cord-stromal, and germ cell neoplasms, where epithelial ovarian tumors constitute the most common form of ovarian carcinomas [1,2]. In addition, non-epithelial ovarian cancers represent 10–15% of all ovarian cancers and include diverse tumors of germ cell origin, sex cord-stromal cell origin, as well as extremely rare types of ovarian cancers, such as small-cell carcinomas and sarcomas [3]. In platinum-sensitive OC patients or those who recur ≥6 months from the initial diagnosis of the disease, treatment has traditionally consisted of platinum-based chemotherapy. Unfortunately, there is a considerable number of patients who show resistance to platinum-based chemotherapy and so current research efforts are focused on the discovery of new therapeutic options. The majority of advanced-stage OC patients, even those who respond well to frontline therapy, will ultimately recur and succumb to their disease [1,2]. Consequently, new therapeutic options are required targeting the discovery of new drug compounds. To this end, the PI3K/AKT/mTOR signaling pathway is one of the most thoroughly investigated ones as it is implicated in several cellular cascades, including growth, motility, survival, proliferation, protein synthesis, autophagy, transcription, as well as angiogenesis. Its deregulation has been shown in several tumors, including ovarian cancer. In this setting, targeted inhibitors have been introduced, while clinical trials have assessed their safety in ovarian cancer patients. Meanwhile, the ongoing phase I and II studies are evaluating the oncologic outcome of these compounds in combination with conventional chemotherapy [4,5].

The discovery of inhibitor molecules of the mammalian rapamycin (mTOR), poly-ADP-ribose polymerase (PARP), MAPK/ERK, and components of the epidermal growth factor receptor (EGFR) pathway are on the frontline of clinical research. In ovarian cancer, important hormones using the extracellular signal-regulated kinase (ERK) pathway (e.g., gonadotropins and gonadotropin-releasing hormones) activate the KRAS-BRAF-MEK axis, and consecutively, ERK which can then activate transcription factors, including myc or elk-1. On the other hand, the mitogen-activated protein kinase (MAPK) pathway develops oncogenic potential through permanent signaling activity as a result of the Kirsten rat sarcoma viral oncogene (KRAS) or v-raf murine sarcoma viral oncogene homolog B (BRAF) mutations, thus inducing cell proliferation. In addition, activation of ERK can activate downstream protein kinases or transcription factors that enhance tumor development [2]. Overall, the MAPK/ERK pathway can contribute to therapy-induced tumor-growth suppression. For instance, ERK-mediated mitogen-activated protein kinase (MAPK) phosphatase-1 (MKP-1) expression is involved in cisplatin resistance, and thus, its targeting could potentially overcome cisplatin resistance in patients with ovarian cancer [6]. Two newly synthesized homo-aza-steroidal alkylating esters of the p-bis-(2-chloroethyl) aminophenoxy-propionic acid (POPAM) were designed in an attempt to improve the therapeutic efficacy in tumors resistant to classical and non-classical alkylating drugs. In general, the lactam steroid alkylators combine two active compounds in a single molecule by incorporating modified steroids, that bear a lactam (–NH-CO-) chemical moiety in one or more steroid rings, and thus functioning as cytotoxic anticancer agents. These hybrid aza-steroid alkylators produce more potent antitumor effects, depending on the alkylating moiety, as well as on the modified steroidal carrier bearing the lactam group, thus increasing anticancer activity significantly and improving the therapeutic ratio while reducing overall systemic toxicity. Finally, these molecules produce a multi-targeted antitumor effect by inhibiting various cellular cascades important for the survival of cancer cells [7,8,9,10,11,12].

In the present study, we have evaluated the cytostatic and cytotoxic effect of two new aza-steroid alkylators (ENGA-L06E and ENGA-L08E) against aniline-mustard alkylators POPAM and melphalan (L-PAM) in a panel of five human ovarian cancer cell lines, as well as on two human ovarian tumor xenograft models. More specifically, we have investigated their inhibitory effects on the RAS/PI3K/AKT and RAS/RAF/MEK/ERK signaling pathways and whether their dual inhibition can potentiate antitumor efficacy by utilizing in silico computational methods.

## 2. Results

We have tested the new hybrid homo-aza-steroidal alkylators against five human ovarian adenocarcinoma cell lines, in vitro, in comparison with the azasteroid esters’ alkylating moiety POPAM. The two conjugates, ENG-L06E and ENGA-L08E, displayed significantly higher anticancer activity, cytostatic, and cytotoxic effects on the tested cell lines than the non-steroidal alkylator POPAM. ENGA-L06E demonstrated rather more potent, cytostatic, and cytotoxic effects than the ENGA-L08E. Furthermore, *TP53* mutations, in all human ovarian cancer cell lines, didn’t affect the cytostatic or cytotoxic effects of ENG-L06E and ENGA-L08E. Similarly, *KRAS* (in OVCAR-5), *MEK1* (in OVCAR-3), *PIK3CA* (in SK-OV-3) mutations, as well as the mismatch repair (MMR) status didn’t invoke the cytostatic or cytotoxic effects of ENG-L06E and ENGA-L08E significantly. In addition, the cytostatic and cytotoxic impact of ENG-L06E and ENGA-L08E was shown to be independent of the steroid receptor expression in all cell lines (Table 1). Both ENG-L06E and ENGA-L08E were more effective against UWB1.289 cells, by means of exerting significantly higher cytostatic and cytotoxic activity, which are known to be Breast cancer type 1 (BRCA1)-null (mutated, lack wild-type BRCA1 gene), in comparison to the other tested cells bearing either the BRCA1 wild type (w.t) or where at BRAC1 is restored (UWB1.289 + BRCA1 cells). Finally, both ENG-L06E and ENGA-L08E were fairly effective against OVCAR-5 and SK-OV-3 cells (both of which exert low sensitivity to the alkylator cisplatin) as well as OVCAR-3 cells, which are (i) resistant to the aniline mustard alkylator melphalan, (ii) not sensitive to cisplatin and neither to Adriamycin [13,14,15,16] (Table 1).

**Figure 1:**
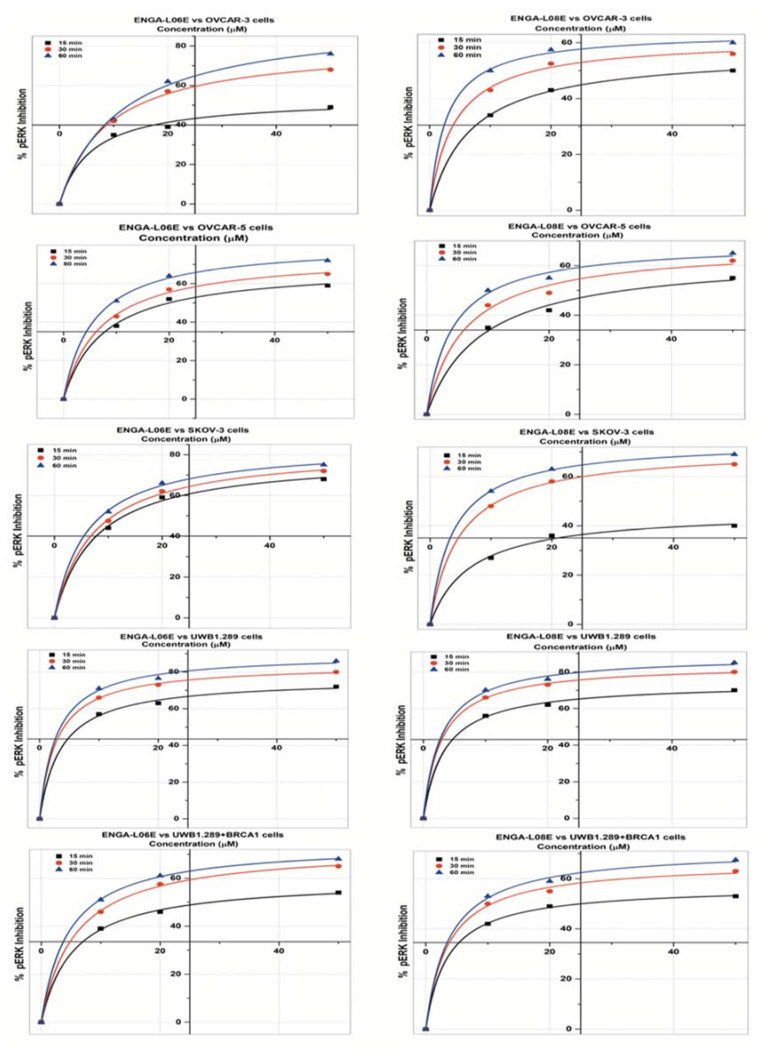
Michaelis Menten model (y = Vmax ∗ x/(Km + x) inhibition curves of the ERK phosphorylation by ENGA-L06E and ENGA-L08E in SKOV-3, OVCAR-3, OVCAR-5, UWB1.289, UWB1.289+BRCA1 human ovarian cancer cell lines, treated with 10, 20, and 50 μM at different time conditions (15, 30, 60 min) (*p* < 0.01).

**Figure 2:**
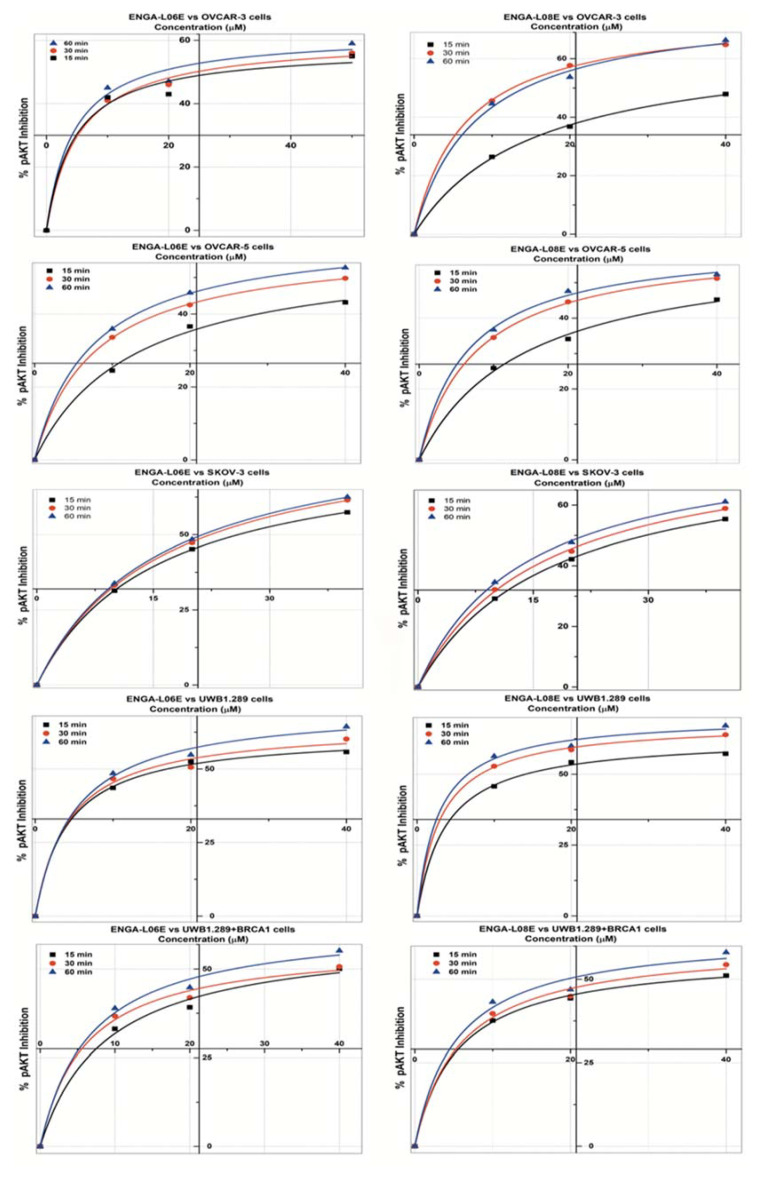
Michaelis Menten model (y = Vmax ∗ x/(Km + x) inhibition curves of AKT phosphorylation by ENGA-L06E and ENGA-L08E in SKOV-3, OVCAR-3, OVCAR-5, UWB1.289, UWB1.289+BRCA1 human ovarian cancer cell lines, treated with 10, 20, and 50 μM at different time conditions (15, 30, 60 min) (*p* < 0.01).

The analytical results of the inhibitory effects of ENGA-L06E and ENGA-L08E on ERK1/2 and AKT1/2 phosphorylation, in all studied cancer cell lines, and the corresponding kinetics according to the Michaelis Menten model are illustrated in Figure 1 and Figure 2, as well as in Table A1 and Table A2. According to the results, both compounds prevented the phosphorylation of ERK1/2 and AKT in all cell lines tested, effectively. The results on pERK1/2 and pAKT inhibitory effect induced by aza-steroidal alkylators ENGA-L06E and ENGA-L08E demonstrated similar patterns of kinetics (Vmax, Km). The highest effect on pERK1/2 inhibition resulted at 60 min and intervened in a dose and time-dependent relevance. In all the tested cell lines, taking together the kinetic parameters and IC50s (the concentrations of tested compounds that induce 50% inhibition of phosphorylation), the ENGA-L06E was shown to be more effective than ENGA-L08E in inhibiting ERK1/2 phosphorylation (Figure 1, Table A1). However, both compounds provoked higher inhibition of pERK1/2 in UWB1.289 cells by about 85% at 60 min. On the other hand, the inhibition of AKT protein phosphorylation caused by ENGA-L08E was somewhat more potent than the inhibitory effect of ENGA-L06E, in all tested cell lines. The highest effect on pAKT inhibition resulted at 60 min and took place in a dose and time-dependent manner, similar to that of ERK1/2 phosphorylation (Figure 2 and Table A2). Overall, ENGA-L08E exhibited a higher inhibitory effect on pAKT in OVCAR-3 cells while ENGA-L06E in SK-OV-3 and UWB1.289 cells (Figure 2). The inhibitory activity of both compounds on ERK1/2 and AKT phosphorylation was not affected significantly by mutations on *TP53*, *PIK3CA*, microsatellite instability (MSI) status, as well as steroid receptor expression and sensitivity to cytotoxic drugs. However, *KRAS* mutation in OVCAR-5 cells may affect AKT phosphorylation, while MEK1 mutations, in OVCAR-3 cells, may derogate the inhibitory effect on ERK1/2 phosphorylation (Table A1 and Table A2). Interestingly, both ENG-L06E and ENGA-L08E show an optimal inhibitory effect, on both ERK1/2 and AKT phosphorylation, in BRCA1-null UWB1.289 cells (Table A1 and Table A2).

**Figure 3:**
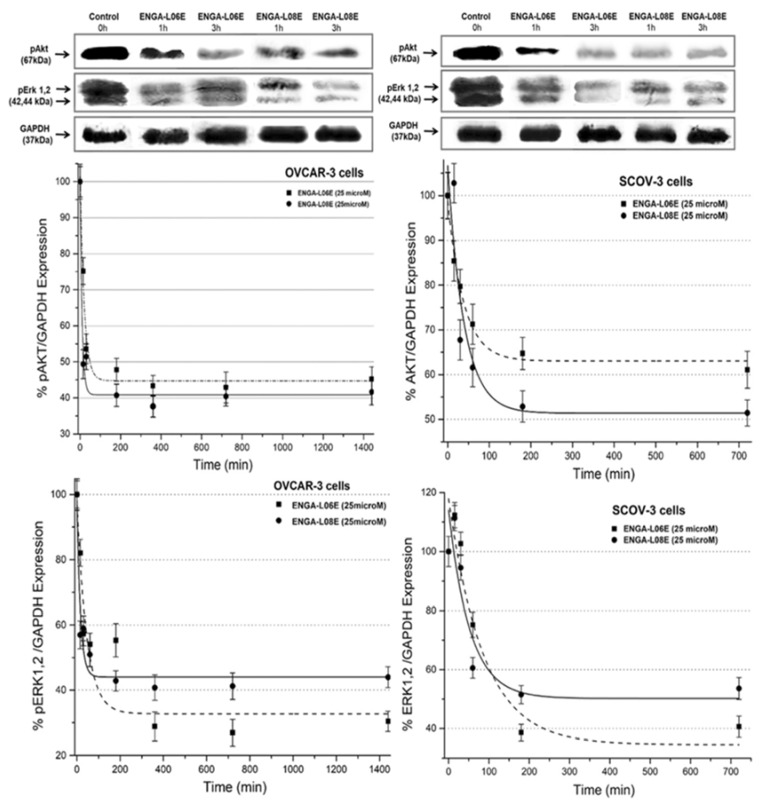
Expression (western blots) and quantification (inhibition curves) levels of ERK1,2 and AKT phosphorylation. Levels of pERK 1,2 and pAKT in SK-OV-3 and OVCAR-3 cell lines after treatment with hybrid aza-steroidal alkylators ENGA-L06E and ENGA-L08E at a concentration of 25 μM in different time intervals. Densitometry readings / intensity ratios of expression and quantification data are presented in Figures S1A,B and S2.

The inhibitory effect of ENGA-L06E and ENGA-L08E on the phosphorylation status of ERK1/2 and AKT, on both ovarian cancer cell lines, is presented in Figure 3. More specifically, the inhibition curves demonstrate t levels of percent expression of ERK1/2/GAPDH and AKT/GAPDH, which reflect more accurately the inhibition of pERK1/2 and pAKT in treated cells. In more detail, the phosphorylation of ERK1/2 and AKT was inhibited under the treatment of both compounds at 25 and 50 μΜ. As the inhibition curves depict, the two tested hybrid molecules yielded approximately 50–70% inhibition at 25 μΜ whilst phosphorylation of targeted proteins was almost totally inhibited (>85%) at higher concentrations (50 μΜ). Moreover, the inhibition of phosphorylation of ERK1/2 and AKT starts in 1 h while it gradually increases in 2 h after which it leads to a plateau (Figure 3). Overall, ENGA-L08E induced a higher inhibition of AKT phosphorylation than ENGA-L06E, whereas ENGA-L06E demonstrated a higher inhibition on ERK1/2 phosphorylation than ENGA-L08E (in both cell lines (Figure 3). Finally, preliminary experimental data (not shown) have shown that the tested azasteroid alkylators inhibit phosphorylation of AKT and ERK in the tumor xenograft tissue in vivo. More detailed quantitative studies on the inhibition of AKT and ERK phosphorylation and the effects on the expression of other predictive biomarkers on tumor cells, such as PD-L1, DNA mismatch repair (MMR) system, in correlation with the treatment schedules, time, and dose administration of the azasteroid alkylators in vivo, are currently under investigation.

ENGA-L06E, ENGA-L08E, L-PAM, and POPAM were evaluated for their in vivo acute toxicity, and such values are illustrated inTable 2. The conjugation of the alkylating agent with the aza-steroidal moiety resulted in significantly reduced acute toxicity effect and an increase of the LD_10_ and LD_50_ values. The LD_50_ and LD_10_ values induced by ENGA-L06E and ENGA-L08E were significantly decreased compared to POPAM and L-PAM. Moreover, ENGA-L08E had a significantly higher acute toxicity effect than ENGA-L06E (Table 2).

**Figure 4:**
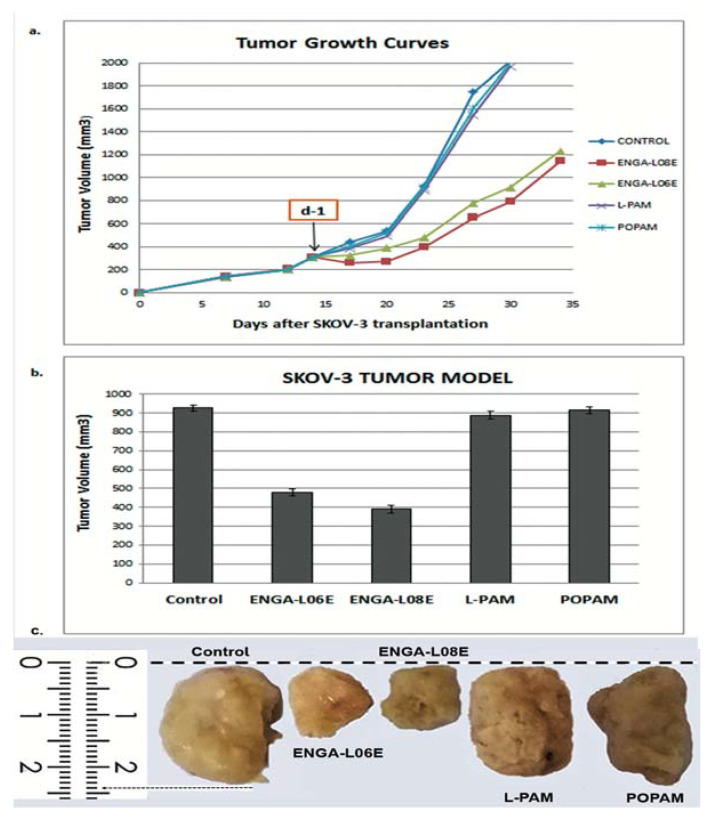
(**a**) In-vivo growth inhibition of the SKOV-3 human ovarian adenocarcinoma induced by ENGA-L06E, ENGA-L08E, L-PAM, and POPAM intraperitoneal administration; (**b**) The tumor volume of the OVCAR-5 adenocarcinoma for all studied compounds on day 9 after drug administration, when there was the highest decrease in tumor volume (induced by ENGA-L08E); (**c**) Representative tumor images of SKOV-3 human ovarian cancer xenograft removed from mice 14 days after treatment administration (day 1) of ENGA-L06, ENGA-L08, L-PAM, and POPAM drugs.

**Figure 5:**
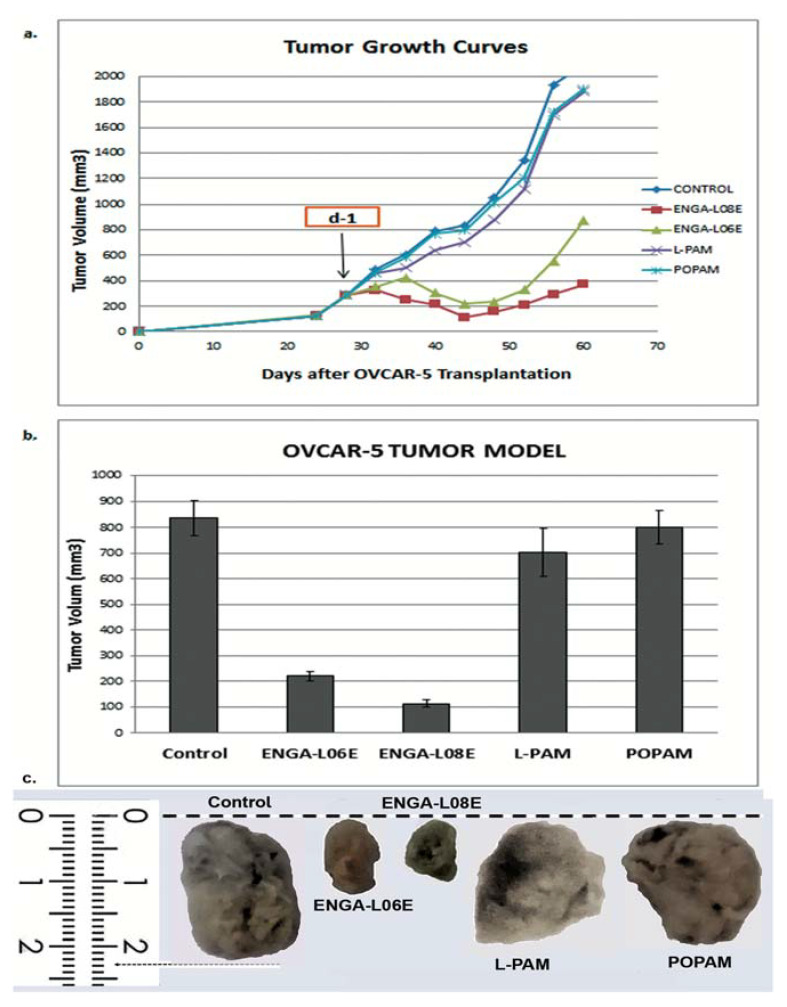
(**a**) In-vivo growth inhibition of OVCAR-5 human ovarian adenocarcinoma induced by ENGA-L06E, ENGA-L08E, L-PAM, and POPAM intraperitoneal administration; (**b**) The tumor volume of the OVCAR-5 adenocarcinoma for all studied compounds, on day 16 after drug administration, when there was the highest decrease in tumor volume (induced by ENGA-L08E); (**c**) Representative tumor images of OVCAR-5 human ovarian cancer xenograft removed from mice 28 days after treatment administration (day 1) of ENGA-L06, ENGA-L08, L-PAM, and POPAM drugs.

In addition, ENGA-L06E and ENGA-L08E were administrated i.p. at the LD_10_ dose and caused significant reduction in the size of SK-OV-3 (Figure 4) and OVCAR-5 (Figure 5) human xenograft tumor in mice. More specifically, 23 and 44 days after implantation, as well as 10 (d-10) and 16 days (d-16) after drug administration, the sizes of the primary tumors were 928 ± 16.4 mm^3^ (Figure 4) and 836 ± 67.4 mm^3^ (Figure 5) for the control group compared to 390 ± 21.4 mm^3^ and 478 ± 18.3 mm^3^ (Figure 4), as well as 113 ± 14 mm^3^ and 220 ± 19.1 mm^3^ (Figure 5) for the groups receiving ENGA-L08E and ENGA-L06E, respectively. 

Furthermore, treatment with ENGA-L08E and ENGA-L06E significantly increased the lifespan of SK-OV-3 as well as of OVCAR-5-bearing mice (Table A5 and Table A6). On the other hand, POPAM and L-PAM showed no significant antitumor activity on SK-OV-3 (Table A3) and OVCAR-5-bearing mice (Table A4). Finally, the tumor response and anticancer activity assessment of ENGA-L06E, ENGA-L08E, POPAM, and L-PAM, according to clinical criteria, demonstrated a significant increase of overall survival (OS), progression-free survival (PFS), and 100% response rate (RR) for ENGA-L06E and ENGA-L08E, respectively. On the other hand, treatment with POPAM and L-PAM showed no significant accrual of OS, PFS, and 100% RR in either SK-OV-3 or OVCAR-5-bearing mice (Table A5).

**Figure 6:**
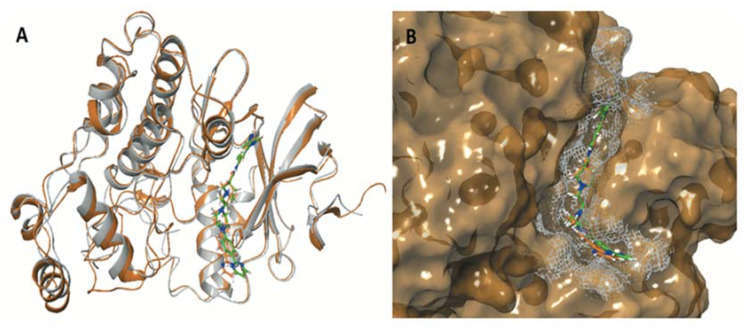
(**A**) Alignment of ERK1 (PDB ID 4QTB) (ribbons with silver) and ERK2 (PDB ID 4QTA) (ribbons with gold) with co-crystallized ligands 38Z0 (green color for ERK1 and orange color for ERK2) and (**B**) Solid surface representation of the crystal structure of ERK1. The crystallographic co-crystallized 38Z0 rendered in green C atoms for ERK1 and the co-crystallized 38Z0 rendered in orange C atoms for ERK2.

The crystallographic structures of the proteins were used during the induced fit docking (IFD) simulations of hybrid steroid organic molecules ENGA-L06E and ENGA-L08E. Their crystal structures were prepared using the Protein Preparation Wizard [17], missing loops and side chains were prepared using Prime, ionized at pH 7.4 using PROPKA [18], and minimized using the OPLS3 force field [19]. The structures were designed using Maestro 10.2 and ionized at the target pH 7.4 using the Ligprep module [20]. Docking simulations were performed in order to elucidate the mechanism of action of the studied compounds at the molecular level, providing an interpretation of their biologic activity as anticancer drugs. The first step was to evaluate the active site for ERK1 and ERK2, a site that is targeted by the same co-crystallized ligand 38Z0, for both proteins (Figure 6A). A grid was generated from the ERK1/2 co-crystallized inhibitor, and the 38Z0 was docked, resulting respectively, in satisfactory overlapping with the crystallographic data. Hydrogen atoms of ligands were omitted for clarity (Figure 6B). ENGA-L06E was found to be less active in both ERK1 and ERK2 than the co-crystalized ligand 38Z0, whereas ENGA-L08E was more active than the ENGA-L06E. In addition, ENGA-L08E bound stronger than the co-crystalized ligand 38Z0 to ERK2 protein. It was also noticeable that in the ERK1 protein, the molecule ENGA-L08E bound efficiently compared to 38Z0 (Table A6). 

**Figure 7:**
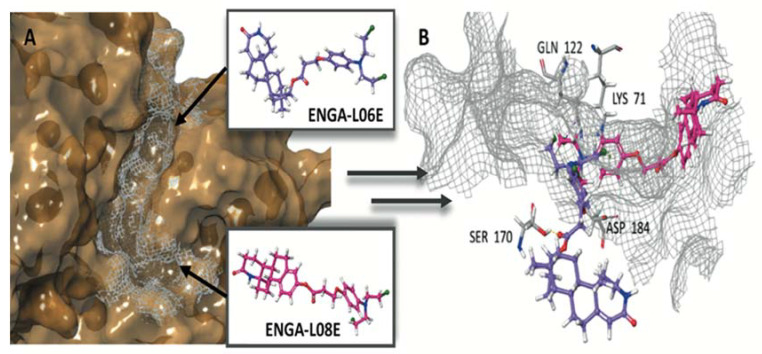
(**A**) The active site cavity of ERK and (**B**) Superimposition of calculated molecules ENGA-L06E and ENGA-L08E given in purple and pink colored carbons, respectively, with labeling of the critical bonding residues.

**Figure 8:**
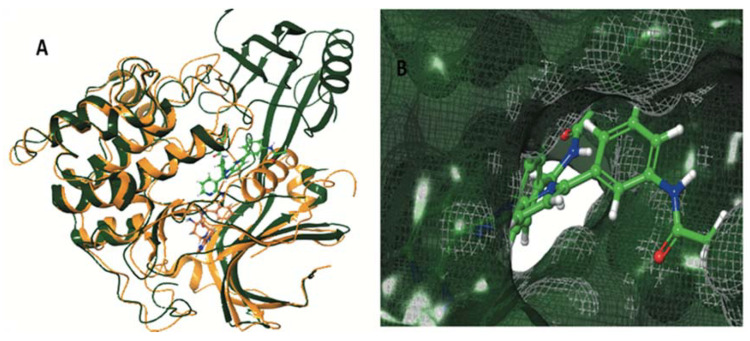
(**A**) Alignment of AKT1 with PDB ID 4EJN (ribbons with dark green) and AKT2 with PDB ID 2X39 (ribbons with yellow) with the co-crystallized inhibitor 0R4 (with green color) and X39 (with yellow color), respectively. (**B**) Surface of AKT1 and the binding site (grey) of the co-crystallized inhibitor 0R4.

**Figure 9:**
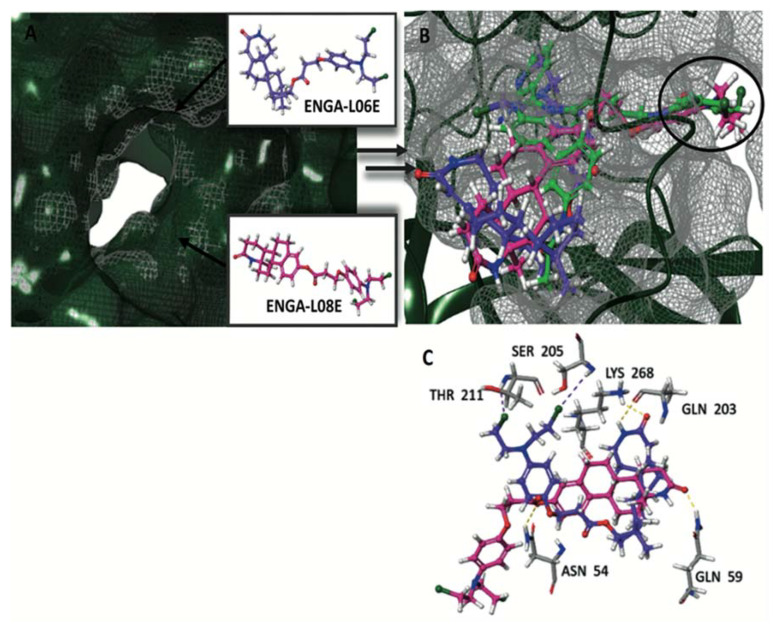
(**A**) The active site cavity of AKT1 and (**B**) Superimposition of the calculated molecules ENGA-L06E and ENGA-L08E, given in purple and pink colored carbons, respectively, with the co-crystallized ligand of AKT1, 0R4 (with green color) and (**C**) The critical network bonding residues are labeled in yellow and purple dashed lines.

Stereochemistry is an important physicochemical property capable of explaining the differences in binding capacity. To this end, ENGA-L08E (with the hot pink color of C atoms) enters the cavity with the same direction as the 38Z0, in contrast to ENGA-L06E that approaches the binding site from the alkylating agent moiety while the steroidal moiety of lactam testosterone remains out of the cavity. Generally, both ENGA-L06E and ENGA-L08E (consisting of a modified steroidal skeleton) came from precursor steroids, testosterone and estrone, respectively, conjugated with the same alkylating agent. The former, due to its aromatic ring and the carboxyl group, which functions as a linker, increase the binding capacity of ENGA-L06E and ENGA-L08E via bonding to residues Lys71 with Halogen and pi-cation bond, and Asp184 with aromatic H-bond. Therefore, the binding efficiency of ENGA-L08E is influenced by the presence of estrone’s aromatic ring in contrast to ENGA-L06E, in which the precursor steroid was testosterone (Figure 7A,B). On the other hand, we evaluated the active site of AKT1 and AKT2 (in the grey grid), which are targeted by two different co-crystallized ligands, 0R4 and X39, respectively. In doing so, we overlapped the two proteins, and AKT1 (the dark green color) was found to be slightly bigger than the AKT2 (the yellow color), while there was a clear difference in the location of the active site where the co-crystallized ligands were located (Figure 8A). In addition to the docking results for both proteins (Table A7), we decided to emphasize on AKT1 protein’s cavity (Figure 8B) given that both ENGA-L06E and ENGA-L08E seem to be inactive for AKT2 (Table A7). The specificity of the active center (Figure 9A), in contrast to the active center of ERK1/2 protein, lies in the fact that it crosses the protein, and in order to inhibit it, the tested molecules should fit into the cavity the same way the co-crystallized ligand 0R4 does (Figure 8B; Table A7).

**Figure 10:**
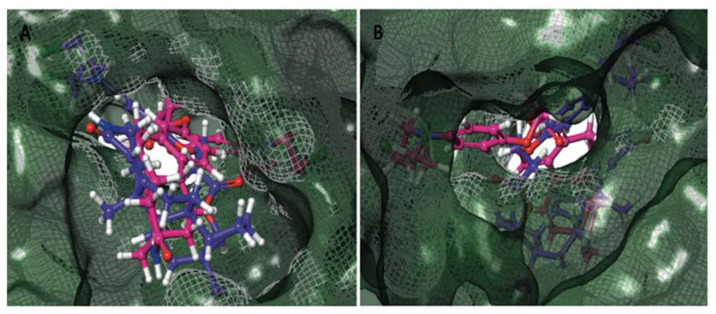
(**A**) Front side and (**B**) Backside of the active site cavity of AKT1 (PDB ID 4EJN) with hybrid molecules ENGA-L06E and ENGA-L08E docked and superimposition of calculated molecules ENGA-L06E and ENGA-L08E given in purple and pink colored carbons, respectively.

After the evaluation of the active site for AKT1, we generated a specific grid for AKT1/2 in a similar way as described above. Subsequently, the hybrid molecules ENGA-L06E and ENGA-L08E were docked in the identified cavity and compared to the co-crystallized ligand OR4. The most active molecule appeared to be ENGA-L08E, whereas ENGA-L06E was also considered as comparable to the co-crystallized ligand (Table A7). A closer view of the docking solution revealed an extended hydrophobic region, as defined by residues Asn54, Gln59, Lys268 for ENGA-L08E and Gln203, Ser205, Thr211, Lys268 for ENGA-L06E (Figure 9C). Although ENGA-L06E further appears to make more contacts with the residues in the active site of the protein, at the same time, it presents slightly higher energy than ENGA-L08E (Table A7) because of the lack of hydrogen bonds when compared to ENGA-L08E (Figure 9C). The superimposition of the three molecules: 0R4 (in green color), ENGA-L06E (in purple color) and ENGA-L08E (in the hot pink color) revealed that the alkylating moiety of ENGA-L08E, and by extension the whole molecule, is precisely adjusted above the co-crystallized ligand in contrast to the ENGA-L06E molecule (Figure 9B). More specifically, in Figure 10A,B, it is clearly represented how the ENGA-L08E molecule is adapted into the AKT1 protein’s cavity. The specificity of this particular active center (Figure 9A), in contrast to the active center of ERK1/2 protein, lies in that it crosses the protein and in order to inhibit it, the tested molecules should fit into the cavity the same way the co-crystallized ligand 0R4 fits (Figure 8B; Table A7). Subsequently, the hybrid molecules ENGA-L06E and ENGA-L08E were docked in the identified cavity and compared to the co-crystallized ligand. The most active molecule appeared to be ENGA-L08E, while ENGA-L06E was also considered comparable to the co-crystalized ligand (Table A7). A closer view of the docking solution in Figure 9C reveals an extended hydrophobic region, as defined by residues Asn54, Gln59, Lys268 for ENGA-L08E, and residues Gln203, Ser205, Thr211, Lys268 for ENGA-L06E. Although ENGA-L06E appears to make more contacts with the residues in the active site of the protein, at the same time, it presents slightly higher energy than ENGA-L08E (Table A7) probably because of the lack of hydrogen bonds (Figure 9C). Complementary to the above data, the superimposition of the three molecules 0R4 (in green color), ENGA-L06E (in purple color) and ENGA-L08E (in the hot pink color) revealed that the alkylating moiety of ENGA-L08E, and by extension the whole molecule, is precisely adjusted above the co-crystalized ligand, in contrast to the ENGA-L06E molecule (Figure 9B). More specifically, from the backside of the protein, it is clearly represented howtheENGA-L08E molecule is adapted into the AKT1 protein’s cavity (Figure 10B).

## 3. Discussion

The new homo-aza-steroidal alkylating esters of POPAM were designed in the context of improving therapeutic efficacy against tumors resistant to alkylating drugs [7,9,21]. Lactam steroid alkylators (LSAs) combine two active compounds in a single molecule, incorporating modified steroids that bear the lactam (–NH-CO-) chemical moiety in one or more steroid rings, with the capacity to act as cytotoxic anticancer agents. These hybrid molecules were aimed to increase anticancer activity, reduce systemic toxicity, and improve the overall therapeutic ratio. Furthermore, LSAs induce a multi-targeted antitumor effect by inhibiting the function of critical cellular pathways important for cancer cell survival cancer cells [7,8,9,10,11,12,22,23,24,25]. 

Of particular importance, RAS/RAF/MEK/ERK and PI3K/PTEN/Akt/mTOR are two signaling pathways capable of regulating a wide array of cellular functions, including proliferation, differentiation, survival, metabolism, and migration. Various studies indicate that AKT and ERK are significantly implicated in cancer cell survival and that their synergism promotes cancer progression and metastasis, thus leading to poor therapeutic outcomes and tumor recurrence. Indeed, RAS/RAF/MEK/ERK and PI3K/PTEN/Akt/mTOR are important pathways. Their activation occurs in approximately 70% of all ovarian cancers. Furthermore, PI3K catalytic subunit alpha (PIK3CA) gene amplification has also been associated with genomic instability, p53 mutation, and a lack of response to chemotherapy. Evidence from ovarian cancer cell lines and animal models revealed that activation of the PI3K/AKT pathway might lead to chemotherapy resistance. Suppression of apoptosis contributed to platinum and taxane resistance. The chemotherapy-induced apoptosis has been restored by specific PI3K inhibitors in vitro and in vivo [26]. Consequently, targeting the regulation of downstream functions by AKT and ERK pathways is of great importance and therapeutic relevance in cancer [27,28]. Even though the downregulation of phosphorylated ERK or AKT may induce cell apoptosis or cell cycle arrest, the crosstalk between these two signaling pathways may counteract the effect of single inhibition. For instance, it has been shown that inhibition of the MEK/ERK pathway enhances AKT phosphorylation while ERK activation attenuates AKT phosphorylation. Therefore, inhibition of either pathway alone may not contribute significantly to the context of an anticancer therapy, whereas inhibition of both pathways may interrupt the transduction of survival signal(s), and consequently, achieve a significant therapeutic effect [29,30]. Previous research has demonstrated that the constitutive activation of AKT in various ovarian cancer cell lines (e.g., SK-OV-3, OVCAR-4, and OVCAR-5) promotes cell survival and resistance to cisplatin. Thus, Akt is a potential therapeutic target as its inhibition might increase cytotoxic sensitivity selectively in ovarian cancer that has a dependence on AKT kinase activity for survival [30]. On the other hand, therapies targeted towards MAPK/ERK components have various response rates in solid tumors, including ovarian cancer. The mechanisms responsible for the acquired chemoresistance in ovarian tumors are many, including efflux pumps, repair mechanisms, signaling survival pathways (e.g., PI3K/AKT, MAPK, estrogen signaling), etc. Specifically, PARP1 inhibition causes a loss of ERK2 stimulation by decreasing the activity of critical pro-angiogenic factors, including the vascular endothelial growth factor (VEGF) and the hypoxia-inducible factor (HIF) [17]. Thus, many natural and synthetic agents capable of suppressing the MAPK/ERK signaling pathway have been tested in order to overcome chemoresistance, and thus a series of targeted inhibitors have emerged [6,31]. Previous studies have shown that LSAs induce an anticancer response through the combination of the direct induction of cellular DNA damage with the inhibition of PARP enzyme(s), and consequently, the inhibition of DNA repair [12,25]. To these ends, inhibitors of nuclear PARP enzymes (e.g., PARP-1) have significantly improved clinical outcomes in ovarian cancer, especially in patients with *BRCA1/2* gene mutations or additional homologous recombination DNA repair pathway deficiencies. Cancers with deficiency of DNA repair (by homologous recombination) are showing greater sensitivity to PARP inhibitors. Beyond BRCA1/2 gene mutations, the loss, perturbation, or overloading of function of various proteins involved in homologous recombination [e.g., ATM, CHEK2, BARD1, BRIP1, MRE11, RAD50, NBS1, RAD51C, RAD51D, RAD52, PALB2, and DNA-dependent protein kinase (DNA-PK)] have been suggested to be synthetically lethal with PARP inhibition. In that case, synthetic lethality could be potentially optimized when PARP inhibitors are combined with DNA-damaging agents, or with molecularly targeted agents capable of impairing DNA repair, such as the case of azasteroid alkylators [32,33].

The response to PARP inhibitors and platinum-based chemotherapy can be predicted by ADP-ribosylation (ADPRylation), which is also catalyzed by PARP enzymes, independently of the *BRCA1/2* or homologous recombination deficiency (HRD) status [34]. In general, PARP-1 is activated by direct interaction with pERK2, which then amplifies ERK-signals, resulting in enhanced pERK2-catalyzed phosphorylation of target transcription factors and increased gene expression. Consequently, the pharmacologic inhibition of PARP can cause loss of ERK2 stimulation, thus blocking the ERK signaling network. Moreover, selective ERK inhibition induces tumor regression in MEK inhibitor-resistant models, and PARP inhibition could be similarly effective in MEK or ERK-resistant tumors. In fact, MEK, ERK, and PARP inhibitors, as well as their combinations, have recently entered in early phase clinical trials [35,36]. On the other hand, given that RAS mutant tumors are usually resistant to PARP inhibitors and other anticancer drugs, the inhibition of MEK and ERK can reverse PARP inhibitor resistance in *KRAS* mutant tumors, including ovarian cancer. Furthermore, the effects of treatment with combinations of PARP and MEK inhibitors are independent of *BRCA1/2* and *p53* mutation status, suggesting a generalized synergistic activity [37]. 

In general, the PI3K/AKT signaling pathway plays a critical role in the therapeutic management of ovarian cancer. More specifically, inhibition of PARP1 activity can lead to the upregulation of the PI3K/AKT pathway, thus promoting resistance to PARP inhibitors (PARPi). For instance, pharmacologic inhibition of PARP-1 promotes PI3K/AKT activity and mTOR signaling resulting in decreased cell death as well as PARPi response. Activation of the AKT survival pathway may counteract the cytotoxic effects of PARP inhibition, suggesting that the AKT pathway inhibition may enhance PARP inhibition in the context of antitumor therapy. As a result, combination(s) of small molecules targeting PARP, as well as the PI3K/AKT pathway, would potentially provide a greater clinical benefit [36,38]. On the other hand, the Ras-ERK and PI3K-mTOR signaling pathways intersect in order to regulate each other and to also co-regulate downstream functions. Both pathways can negatively regulate each other’s activity, and cross-inhibition is often emerging. Moreover, when one pathway is chemically blocked, the other pathway can effectively be activated. Also, the Ras-ERK pathway cross-activates PI3K-AKT-mTORC1 signaling as long as Ras-GTP can directly bind and allosterically activate Phosphoinositide 3-kinase (PI3K). Finally, both pathways are often activated by genetic alterations in the upstream signaling molecules, which could further result in increased transcription of various genes promoting cell growth and malignant transformation. So, the rationale of dual inhibition of both of these pathways in suppressing ovarian cancer appears to be promising [39]. In fact, the extent and importance of this crosstalk and its significance in cancer therapeutics is becoming evident since AKT and ERK signaling are both aberrantly activated in a wide range of other human cancers [40]. Nevertheless, the utilization of dual-targeted inhibitors for PI3K/AKT/mTOR and RAS/ERK signaling pathways is a potentially effective therapeutic approach against ovarian cancer [41].

On the other hand, steroid hormones promote epithelial ovarian cancer growth, and their receptor expression is associated with disease outcome. Estrogen (ER-α/-β), and progesterone receptors PR(-A/-B) are frequently expressed in ovarian cancer with an important variability relating to histological subtype, grade, and stage. Hormone therapy is frequently used, but the significance of activity by specific agents or tumor characteristics is unknown. Although the activity of endocrine therapy in advanced ovarian cancer is considerable, limited clinical trials have shown modest response rates to endocrine therapy. However, a small subset of patients that respond very well was identified with few side effects [42,43,44]. The steroid components of ENGA-L06E and ENGA-L08E are testosterone and estrone derivatives, respectively. It has been reported that the steroidal moiety of homo-aza-steroid alkylators is binding to estrogen receptors (ER-α), in breast cancer cells with positive ER expression, and consequently was shown to be more sensitive to their cytostatic and cytotoxic effects [25,45]. However, both ENGA-L06E and ENGA-L08E compounds were shown significant anticancer activity against all human ovarian carcinoma cell lines tested and regardless of the steroid receptor expression.

## 4. Materials and Methods 

### 4.1. Synthesis of Chemical Compounds

**Figure 11:**
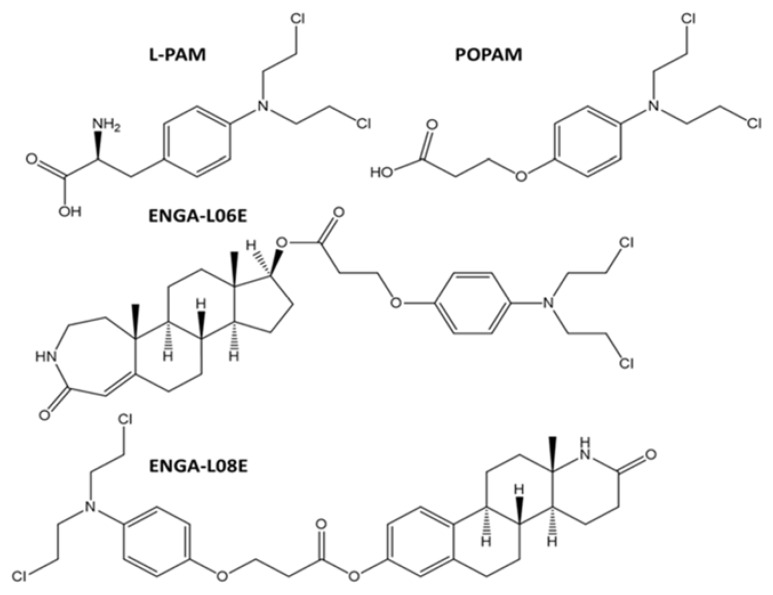
Chemical structures of 2-amino-3-(4-(bis (2-chloroethyl) amino) phenyl) propanoic acid (L-phenylalanine mustard, L-PAM), 3-(4-(bis(2-chloroethyl) amino) phenoxy) propanoic acid (POPAM), 5a,7a-dimethyl-2-oxo-2,3,4,5,5a,5b,6,7,7a,8,9,10,10a,10b,11,12-hexa decahydrocyclopenta [5,6] naphtho [1,2-d] azepin-8-yl 3-(4-(bis(2-chloroethyl)amino) phenoxy) propanoate (ENGA-L06E) and 12a-methyl-2-oxo-1,2,3,4,4a,4b,5,6,10b,11,12,12a-dodecahydronaphtho[2,1-f]quinolin-8-yl 3-(4-(bis (2-chloroethyl)amino) phenoxy) propanoate (ENGA-L08E).

All modified hybrid steroidal esters and comparative alkylators were synthesized with methods that have been previously described [23]. More specifically, we have tested the two previously synthesized hybrid homo-azasteroid alkylators (e.g., ENGA-L06E and ENGA-L08E) along with the proper reference alkylator aniline mustards (e.g., POPAM and L-PAM). The lactam-steroidal carrier of ENGA-L06E is a derivative of testosterone while ENGA-L06E of estrone. The chemical structures of all studied compounds are shown in Figure 11.

### 4.2. Cell Lines

Five human ovarian cancer cell lines (UWB1.289, UWB1.289+BRCA1, SK-OV-3, OVCAR-3, and OVCAR-5) were treated with the newly synthesized compounds at concentrations of 1–100 μΜ in order to test their cytostatic and cytotoxic activity. All cell lines were obtained from the American Type Culture Collection (ATCC), USA except OVCAR-5, which was kindly donated from BRFFA, Academy of Athens, Greece, and were grown in different culture media (according to the manufacturer’s recommendations), 37 °C and 5% CO_2_. Finally, all specific molecular characteristics and mutated genes’ profiles, in all five human ovarian cancer cell lines, concerning chemotherapeutic drug tolerance, are shown in Table A8. The cell lines have been tested with standards procedures for microbial contamination, including mycoplasma.

### 4.3. Determination of Cell Viability by the MTT Assay

Cells were plated in 96-well plate at a density of 1 × 10^4^ cells/mL per well and maintained for 72 h at 37 °C in a 5% CO_2_ incubator and grown as monolayers. After 24 h, cells were treated with 0.1–100 μmol/L of the tested compounds for 48 h. The viability of cultured cells was estimated by utilizing the (3-(4,5-imethylthiazol-2-yl)-2,5-diphenyltetrazolium bromide (MTT) metabolic assay, as described previously [23,24]. Briefly, MTT (Sigma, St Louis, Missouri, USA) was dissolved in PBS in a concentration of 5 mg/mL, filter sterilized, and stored at 4 °C. Then, 50 μL of stock solution was added to each culture and incubated for 3 h at 37 °C. Formazan crystals were solubilized by DMSO (100 μL). The absorbance of the converted dye was measured at a wavelength of 540 nm on ELISA reader (VersaMax Microplate Reader Orleans, USA). The mean concentrations of each drug that generated 50% or total (100%) growth inhibition (GI_50_ and TGI, respectively), as well as the drug concentrations that produced cytotoxicity against 50% of the cultured cells [half maximal inhibitory concentration (IC_50_)], were calculated using the linear regression method [46]. Using seven absorbance measurements [time 24 h (Ct24), control growth 72 h (Ct72), and test growth in the presence of the drug at five concentration levels (Tt72x)], the percentage of growth was calculated at each level of the drug concentrations. The percentage growth inhibition was calculated according to National Cancer Institute (NCI) as: [(Tt72x) − (Ct24)/(Ct72) − (Ct24)] × 100 for concentrations for which Tt72x > Ct24 and [(Tt72x) − (Ct24)/Ct24] × 100 for concentrations for which Tt72x < Ct24. The GI_50_ was calculated from [(Tt72x) − (Ct24)/(Ct72) − (Ct24)] × 100 = 50 while the TGI from [(Tt72x) − (Ct24)/(Ct72) − (Ct24)] × 100 = 0, and IC_50_ from [(Tt72x) − (Ct24)/Ct24] × 100 = 50. All experiments were carried out in triplicate.

### 4.4. Determination of ERK 1/2 and AKT 1/2/3 Phosphorylation by ELISA

Phosphorylation of ERK 1/2 (Thr202/Tyr204, Thr185/Tyr187) and AKT 1/2/3 (Ser473) was determined by the Activation InstantOne™ ELISA assay kit (Invitrogen, eBioscience, (San Diego, CA, USA) at 450 nm and according to manufacturer’s recommendations. All cell lines were cultured in 12-well plates, seeding 10^5^cells per well. After 24 h, cells were treated with ENGA-L06E and ENGA-L08E, at concentrations of 10, 20, and 60 μΜ, and time exposures of 0, 15, 30, and 60 min. Cells were washed in PBS, extracted in RIPA buffer, and the protein content was quantified by the Bradford assay. Then, 50 μg/mL of protein concentration was added in a 96-well plate, in triplicates, followed by the addition of 50 μL/well of mixed antibodies AKT/ERK 1,2 and pAKT/ERK 1,2 for 1 h. Samples were washed three times, and then 100 μL/well of detection reagent was added and incubated for 30 min. Finally, the reaction was terminated by the addition of 100 μL/well of STOP solution, after which the absorption of all samples was measured at 450 nm.

### 4.5. Determination of ERK 1/2 and AKT 1/2/3 Phosphorylation by Western Immunoblotting

In order to confirm that ENGA-L06E and ENGA-L08E inhibit the phosphorylation of ERK1/2 and AKT 1/2/3, western immunoblotting was utilized [46]. Cells were treated with the tested compounds for 0, 0.25, 0.5, 1, 3, 6, 12, and 24 h, and samples were prepared in the presence of ice-cold RIPA buffer and protease inhibitor cocktail mixture. The proteinaceous extract was vortexed, centrifuged at 14,000× *g*, for 10 min, at 4 °C and the total protein content was quantitated by the BCA assay. Then, an equal quantity (25 μg) from each protein sample was loaded into SDS-PAGE electrophoresis followed by transfer to a nitrocellulose membrane with further incubation at 5% skim milk in Tween 20-Tris buffered saline (TBST). After blocking, the membrane was incubated overnight with primary antibodies [ERK1/2, pERK1/2, pAKT, AKT, and Glyceraldehyde 3-phosphate dehydrogenase (GAPDH] at specified concentrations. Finally, the membrane was washed and then incubated with the HRP-conjugated rabbit anti-mouse secondary antibody. Signals were detected using an automated image acquisition system, while all acquired images were further normalized to GAPDH and semi-quantified by the ImageJ software (NIH, Bethesda, Washington D.C., USA).

### 4.6. Determination of In Vivo Toxicity and Antitumor Efficacy

CB17 severe combined immunodeficiency (SCID) male and female mice were obtained from the National Center for Scientific Research (NCSR) Demokritos, Institute of Biology, Athens, Greece. They were fed water and an irradiated standard rodent diet *ad libitum* and housed under pathogen-free conditions. All animals were sacrificed by cervical disruption when shown signs of cachexia, suffering, or the tumor volume reached 2000 mm^3^. For intraperitoneal (i.p.) treatment, stock solutions of the four tested compounds were prepared immediately before use. They were suspended in corn oil, in the desired concentration, following initial dissolution in 10% dimethylsulfoxide (DMSO) without a record of any observable toxic effects. Briefly, acute toxicity induced by the tested compounds was determined after a single i.p. injection (into groups of ten CB17 SCID mice, which were then observed for 30 days) and as described previously [11,47]. The LD_10_ and LD_50_ (lethal dose for 10% and 50% of animals, respectively) were determined after graphical estimation (30-day curves) where the percentage of deaths due to the toxicity of each dose was shown in the ordinate while the administered doses were indicated on the abscissa. Two animal models were established based on either SK-OV-3 or OVCAR-5 being implanted subcutaneously (s.c.) into CB17 SCID mice. Overall, at Day 0 (D0), 4 × 10^6^ cells were implanted s.c., and mice were randomized into either test (*n* = 5) or control (*n* = 7) groups while their tumor volumes were in the 250–300 mm^3^ range. After randomization, all animals were treated i.p. with either the tested compounds (at a single LD_10_ dose; test group) or saline buffer (at a single 0.2 mL dose; control group). The first day that each animal model was treated with each of the tested compounds was determined as Day 1 (d-1). Finally, all stock solutions for each of the tested compounds were prepared immediately before use. The subcutaneously tumors were measured, and the animals were weighed twice a week starting on the first day of treatment. Tumors were measured using an analog caliper. Tumor volume (mm^3^) was calculated according to the formula D × d^2^/2, where D and d refer to the larger and smaller perpendicular dimensions collected at each measurement, respectively. The diameters were determined from two orthogonal measurements on each tumor. The mean tumor volume was determined by average, all tumor volumes in each group [48]. On the other hand, for the evaluation of antitumor efficacy, the following parameters were considered: (i) weekly mean tumor area change and tumor inhibition (TI) were determined by utilizing the formula: TI(%) = [1-(TWT − TWZ)/(TWC − TWZ)] × 100 [where TWT is the tumor area (mm^3^) in treated animals at the time of evaluation; TWZ is the tumor area (mm^3^) at the time of initiation of treatment (day-1), and TWC is the tumor volume (mm^3^) in untreated animals (controls) at the time of evaluation]; (ii) percentage of increase in median lifespan [(T/C)%] of treated (T) over control (C) animals. All mice were sacrificed when the tumor volume had reached 2000mm^3^ (endpoint) [47,49]. Furthermore, three other clinical criteria were also considered as part of our final determination(s): (iii) time of progression-free survival (PFS) which represents the time, in days, that tumor volume presented growth >20% (after treatment with each test compound); (iv) overall survival (OS), which represents the median time, in days [where the treated and untreated animals lived from the day of tumor cells inoculation (D0)]; (v) response rate (RR) which represents the percentage of dose-related response to the tested compounds, as it is interpreted by tumor volume regression (>50%).

The protocol was approved by the ethical committee of the National & Kapodistrian University of Athens and was conducted according to the European Directive 86/609/EEC guidelines for the care and use of laboratory animals. The study received a permit from the Veterinary Directorate of the Prefecture of Athens (Approval #: 2023/2017) according to the Greek legislation conforming to the 2010/53/EU Council Directive.

### 4.7. Elucidation of Underlined Molecular Mechanism(s) by in Silico Approaches

Crystal structures of AKT1, AKT2, ERK1, and ERK2 were available in the RCSB Protein Data Bank of Brookhaven National Laboratory from which we have chosen the ones with the highest resolution (Å) (the higher the resolution, the lower the Å value). The chosen PDB carries a co-crystalized ligand with a carbon chain quite large to resemble as much as possible with our long chain steroid hybrid molecules in order to fit properly at the proteins’ binding site cavity. These structures were superimposed, showing no conformational variability for the backbone and for most sidechains. Waters were deleted with Maestro, the graphical interface of Schrödinger software (http://www.schrodinger.com), prior to docking. Molecular docking studies were carried out for the best-fitted compounds to the model, while the final selection criteria were compounds docking scores and the presence of crucial interactions for binding to the studied proteins [50]. Finally, the resulting poses were examined manually, and the most promising ones were used again in docking with Induced Fit Docking protocol 2013-2 [51,52], Glide, version 5.9, Prime, version 3.2 (Schrödinger, LLC, New York, NY) [53,54]. To rationally design the steroidal conjugates to be able to bind to the binding sites of ERK1/2 and AKT1/2, we used for the induced fit docking (IFD) process the different available crystal structures shown in Table A9. All of our newly synthesized compounds are sketched and converted into three-dimensional MOL2 files using Schrödinger Release 2015-2, Maestro Version 10.5, and minimized using Schrödinger Release 2015-2, LigPrep 3.4 [54], and the OPLS3 [20] force field. In all cases, the protonation state for the compounds was adjusted at physiological pH using LigPrep (Schrödinger, http://www.schrodinger.com).

### 4.8. Statistical Analysis

All experiments were repeated in triplicate. Data were presented as mean ± standard deviation (SD). Statistical differences between experimental groups were compared using the Student’s *t*-test (two-tailed paired). Statistical significance was considered at *p* < 0.05. Microsoft Excell (Microsoft Hellas, Athens, Greece) was used to analyze the data.

## 5. Conclusions

Two new homo-aza-steroidal alkylating ester compounds (e.g., ENGA-L06E and ENGA-L08E) were tested in an in vitro model of human ovarian carcinoma (consisting of five cell lines namely UWB1.289, UWB1.289+BRCA1, SK-OV-3, OVCAR-5, and OVCAR-3) as well as an in vivo model consisting of SK-OV-3 and OVCAR-5 tumor xenografts. Both compounds were found to exert very high anticancer activity by inhibiting both the PI3K-AKT and KRAS-ERK signaling pathways. Detailed in silico computational studies were also in accordance and confirmed results of the in vitro studies. As has been previously reported, both of these compounds are capable of inhibiting PARP activity, and consequently, overcome resistance of ovarian cancer cells to alkylating agents, including cisplatin. Moreover, mutations on *TP53, KRAS, MEK1* and *PIK3CA*, microsatellite instability (MSI) status, and steroid receptor expression did not affect the anticancer effect of both compounds. Thus, lactam steroidal alkylators (e.g., ENGA-L06E and ENGA-L08E) provide a novel combination of pharmacological properties exerting significant antitumor activity against human ovarian carcinoma independently of the RAS-ERK and PI3K-AKT mutation, MMR and steroid receptor status, as well as, existing tumor cell resistance to alkylating agents. Conclusively, these data support evidence for further development of lactam steroid alkylators in clinical studies on the treatment of epithelial ovarian carcinoma.

## Figures and Tables

**Table 1:** In vitro growth inhibition/cytostatic [50% Growth Inhibition (GI_50_) and Total Growth Inhibition (TGI) μΜ] and cytotoxic effects (IC_50_ μM) of compounds POPAM, ENGA-L06E, and ENGA-L08E on OVCAR-3, OVCAR-5, SK-OV-3, UBW 1.289, and UWB1.289+BRCA1 cancer cell lines.

	POPAM			ENGA-L06E			ENGA-L08E		
	GI50	TGI	IC50	GI50	TGI	IC50	GI50	TGI	IC50
OVCAR-3	56 ± 2.7	92 ± 7.5	>100	22 ± 0.9	32 ± 1.8	43 ± 2.9	30 ± 2.1	40 ± 2.7	56 ± 3.2
OVCAR-5	52 ± 2.3	87 ± 7.8	>100	25 ± 1.4	42 ± 2.8	65 ± 3.4	11 ± 0.6	60 ± 2.7	92 ± 8.4
SK-OV-3	50 ± 2.9	>100	>100	18 ± 0.9	52 ± 3.3	76 ± 7.5	10 ± 0.5	76 ± 7.2	>100
UWB1.289	22 ± 1.9	86 ± 7.4	>100	3.5 ± 0.4	8 ± 0.8	20 ± 1.1	8 ± 0.9	20 ± 1.5	45 ± 3.3
UWB1.289+BRCA1	>100	>100	>100	12 ± 0.7	28 ± 1.9	40 ± 3.5	18 ± 1.0	76 ± 7.2	88 ± 8.1

**Table 2:** Acute toxicity of the compounds L-PAM, POPAM, ENGA-L06E, ENGA-L08E in CB17 SCID mice, where LD_50_ and LD_10_ are the lethal doses for 50% and 10% of the treated mice population.

Compounds	LD50 (mg/kg)	LD10 (mg/kg)
L-PAM	20.8	14
POPAM	20	15
ENGA-L06E	150	120
ENGA-L08E	95	75

## Supplementary Materials

The following are available online at https://www.mdpi.com/2072-6694/12/5/1263/s1, Figure S1: Densitometry readings / intensity ratios of expression and quantification data of Figure 3, Figure S2: Whole western blots, with molecular weight markers (M.W.M.), presenting the expression levels of pERK 1,2 and pAKT in SK-OV-3 and OVCAR-3 ovarian cancer cell lines after treatment with hybrid aza-steroidal alkylators ENGA-L06E and ENGA-L08E at a concentration of 25 μM in 1 and 3 h intervals.

## Appendix A

**Table A1:** The results of inhibitory effects of ENGA-L06E and ENGA-L08E on ERK1,2 phosphorylation, in all studied cancer cell lines, as well as the corresponding kinetics according to Michaelis Menten model (significance level *p* < 0.01).

pERK 1/2 INHIBITION Model Michaelis Menten/Equation: y = Vmax ∗ x/(Km + x)						
ENGA-L06E				ENGA-L08E		
**OVCAR-5 cells**	15 min	30 min	60 min	15 min	30 min	60 min
Reduc. Chi-Sqr	3.126	1.950	0.867	3.435	7.354	3.869
Adj. R-Square	0.995	0.998	0.999	0.994	0.990	0.995
Vmax ± SE	68.95 ± 3.21	74.99 ± 2.47	80.67 ± 1.52	63.85 ± 3.62	67.94 ± 4.54	69.19 ± 2.95
Km ± SE	7.58 ± 1.33	7.06 ± 0.91	5.64 ± 0.48	8.97 ± 1.75	6.09 ± 1.74	4.17 ± 0.97
IC50 (μM)	20.02	14.12	9.18	32.38	16.96	10.86
**OVCAR-3 cells**						
Reduc. Chi-Sqr	4.244	1.035	2.725	0.081	1.600	0.941
Adj. R-Square	0.991	0.999	0.998	0.9998	0.998	0.999
Vmax ± SE	53.57 ± 3.41	80.62 ± 1.98	93.91 ± 3.61	56.77 ± 0.49	61.29 ± 1.88	63.79 ± 1.33
Km ± SE	5.91 ± 1.64	8.87 ± 0.75	11.21 ± 1.32	6.61 ± 0.23	4.01 ± 0.69	2.63 ± 0.42
IC50 (μM)	49	14.47	12.76	48.85	17.77	9.51
**SK-OV-3 cells**						
Reduc. Chi-Sqr	2.074	0.895	0.906	1.449	0.126	0.086
Adj. R-Square	0.998	0.999	0.999	0.996	0.9998	0.9999
Vmax ± SE	79.14 ± 2.62	82.95 ± 1.69	84.71 ± 1.60	45.92 ± 2.07	71.49 ± 0.55	74.32 ± 0.43
Km ± SE	7.59 ± 0.95	7.23 ± 0.57	6.10 ± 0.49	6.52 ± 1.20	4.83 ± 0.19	3.72 ± 0.13
IC50 (μM)	13.01	10.97	8.78	>50	11.25	7.67
**UWB1.289 cells**						
Reduc. Chi-Sqr	2.119	0.404	3.360	1.322	0.404	2.372
Adj. R-Square	0.998	0.9997	0.998	0.9987	0.9997	0.99844
Vmax ± SE	76.21 ± 2.11	84.08 ± 0.88	89.63 ± 2.53	73.97 ± 1.64	84.08 ± 0.88	88.83 ± 2.13
Km ± SE	3.57 ± 0.60	2.81 ± 0.21	2.80 ± 0.57	3.36 ± 0.47	2.81 ± 0.21	2.84 ± 0.49
IC50 (μM)	6.81	4.12	3.53	7.02	4.25	3.67
**UWB1.289+BRCA1 cells**						
Reduc. Chi-Sqr	0.465	0.483	0.101	0.181	1.908	1.626
Adj. R-Square	0.999	0.999	0.9998	0.9997	0.998	0.998
Vmax ± SE	59.43 ± 1.10	72.79 ± 1.14	74.35 ± 0.49	56.97 ± 0.61	66.56 ± 1.99	71.72 ± 1.86
Km ± SE	5.41 ± 0.46	5.67 ± 0.40	4.52 ± 0.15	3.49 ± 0.23	3.53 ± 0.65	3.72 ± 0.57
IC50 (μM)	28.68	12.43	9.29	25.02	10.65	8.56

**Table A2:** The results of inhibitory effects of ENGA-L06E and ENGA-L08E on AKT1,2 phosphorylation, in all studied cancer cell lines, as well as the corresponding kinetics according to Michaelis Menten model (significance level *p* < 0.01).

pAKT 1/2 INHIBITION Model Michaelis Menten/Equation: y = Vmax ∗ x/(Km + x)						
ENGA-L06E				ENGA-L08E		
**OVCAR-5 cells**	15 min	30 min	60 min	15 min	30 min	60 min
Reduc. Chi-Sqr	0.429	0.321	2.198	1.376	0.086	1.047
Adj. R-Square	0.999	0.999	0.996	0.996	0.9998	0.998
Vmax ± SE	57.76 ± 3.49	59.24 ± 0.49	62.49 ± 0.25	60.45 ± 3.65	61.17 ± 0.62	61.47 ± 2.03
Km ± SE	12.77 ± 2.12	7.71 ± 0.23	7.37 ± 0.11	14.15 ± 2.24	7.64 ± 0.28	6.48 ± 0.85
IC50 (μM)	>50	49.84	29.51	>50	34.19	28.26
**OVCAR-3 cells**						
Reduc. Chi-Sqr	11.207	3.445	12.978	0.233	0.250	3.633
Adj. R-Square	0.983	0.994	0.978	0.999	0.9997	0.996
Vmax ± SE	57.71 ± 5.51	60.76 ± 2.97	62.20 ± 5.12	65.97 ± 1.60	75.64 ± 0.99	78.39 ± 4.18
Km ± SE	4.49 ± 2.22	5.25 ± 1.20	4.47 ± 1.91	15.34 ± 0.94	6.48 ± 0.34	8.06 ± 1.51
IC50 (μM)	29.13	24.42	18.31	>50	12.75	14.19
**SK-OV-3 cells**						
Reduc. Chi-Sqr	0.014	0.1064	0.030	1.848	2.867	2.637
Adj. R-Square	0.9999	0.9998	0.9999	0.996	0.995	0.996
Vmax ± SE	79.23 ± 0.39	86.10 ± 1.13	87.27 ± 0.59	79.22 ± 0.89	81.78 ± 2.53	82.20 ± 1.68
Km ± SE	15.16 ± 0.19	16.13 ± 0.52	15.92 ± 0.27	17.31 ± 0.46	15.85 ± 1.21	14.03 ± 0.76
IC50 (μM)	25.94	22.34	21.37	29.62	24.94	21.78
**UWB1.289 cells**						
Reduc. Chi-Sqr	0.702	6.108	3.483	0.670	0.409	3.296
Adj. R-Square	0.999	0.992	0.996	0.999	0.9995	0.997
Vmax ± SE	62.12 ± 1.41	64.94 ± 4.22	71.12 ± 3.34	63.02 ± 1.33	68.23 ± 0.99	70.28 ± 2.75
Km ± SE	4.11 ± 0.50	4.31 ± 1.45	4.98 ± 1.10	3.67 ± 0.45	3.00 ± 0.29	2.68 ± 0.77
IC50 (μM)	16.93	14.41	11.77	14.07	8.24	6.60
**UWB1.289+BRCA1 cells**						
Reduc. Chi-Sqr	4.130	3.108	4.314	0.587	4.069	8.907
Adj. R-Square	0.991	0.994	0.993	0.999	0.993	0.986
Vmax ± SE	59.78 ± 4.67	57.10 ± 3.38	63.12 ± 4.19	57.59 ± 1.42	60.90 ± 3.82	63.84 ± 5.49
Km ± SE	8.81 ± 2.30	6.00 ± 1.49	6.76 ± 1.75	5.49 ± 0.60	5.80 ± 1.55	5.37 ± 2.07
IC50 (μM)	44.42	40.26	25.78	36.23	26.62	19.42

**Table A3:** In vivo antitumor effects of compounds ENGA-L06E, ENGA-L08E, L-PAM and POPAM on SKOV-3 human ovarian adenocarcinoma xenograft bearing mice.

Compounds	Treatment Schedule (Days)	Dose (mg/kg)	MST ^a^ (Days)	TI% ^b^	T/C% ^c^	Cures ^d^
ENGA-L08E	d-1	75 (i.p.)	76.5 ± 3.9 *p* < 0.01	76.4	187.9	2/5
ENGA-L06E	d-1	120 (i.p.)	79.6 ± 4.0 *p* < 0.01	67.6	195.6	2/5
L-PAM	d-1	14 (i.p.)	48.5 ± 2.3	10.5	119.7	0/5
POPAM	d-1	15 (i.p.)	47.7 ± 1.5	12.0	117.2	0/5
Controls	d-1	saline	40.7 ± 3.0	--	100.0	0/7

Tumor cells were inoculated at day 0; ^a^ MST: mean survival time (days), animals were sacrificed when the tumor volume had reached 2000mm^3^ (endpoint); ^b^ TI: % Tumor Inhibition; ^c^ T/C: mean survival time (%) of drug-treated animals (T) versus saline-treated controls (C); ^d^ Cures: Defined as mice alive for 120 days after tumor inoculation (D0).

**Table A4:** In vivo antitumor effects of compounds ENGA-L06E, ENGA-L08E, L-PAM and POPAM on OVCAR-5 human ovarian adenocarcinoma xenograft bearing mice.

Compounds	Treatment Schedule (Days)	Dose (mg/kg)	MST ^a^ (Days)	TI% ^b^	T/C% ^c^	Cures ^d^
ENGA-L08	d-1	75 (i.p.)	94.8 ± 6.3 *p* < 0.01	131.0	187.8	3/5
ENGA-L06E	d-1	120 (i.p.)	93.2 ± 2.4 *p* < 0.01	117.0	184.8	0/5
L-PAM	d-1	14 (i.p.)	61.0 ± 4.3	24.5	120.8	0/5
POPAM	d-1	14 (i.p.)	59.7 ± 3.3	6.22	118.3	0/5
Controls	d-1	saline	50.5 ± 5.8	--	100.0	0/7

Tumor cells were inoculated at day 0; ^a^ MST: mean survival time (days), animals were sacrificed when the tumor volume had reached 2000 mm^3^ (endpoint); ^b^ TI: % Tumor Inhibition; ^c^ T/C: mean survival time (%) of drug-treated animals (T) versus saline-treated controls (C); ^d^ Cures: Defined as mice alive for 120 days after tumor inoculation (D0).

**Table A5:** Evaluation of the in vivo antitumor effect with the clinical criteria: Median Overall Survival (OS), Progression Free Survival (PFS) and Response Rate (RR).

	Clinical Research Criteria					
	Median Overall Survival (OS) (Days)		Progression Free Survival (PFS) (Days)		Response Rate (%) (RR)	
Drugs	SK-OV-3	OVCAR-5	SK-OV-3	OVCAR-5	SK-OV-3	OVCAR-5
ENGA-L06	112	98	11	27	100	100
ENGA-L08	105	127	13	34	100	100
L-PAM	49	60	0	0	0	0
POPAM	49	57	0	0	0	0
Control	50 (>2000 mm^3^)	40	0	0	0	0

**Table A6:** Docking scores and Glide e-model in kcal/ mol of molecules ENGA-L06E and ENGA-L08E compared to the co-crystal ligand 38Z0 in proteins ERK1 and ERK2.

	ERK1 (pdb ID: 4QTB)		ERK2 (pdb ID: 4QTA)	
	Docking Score (kcal mol^−1^)	Glide e-Model (kcal mol^−1^)	Docking Score (kcal mol^−1^)	Glide e-Model (kcal mol^−1^)
Co-crystalized ligand 38Z0	−10.372	−129.250	−8.244	−104.505
ENGA-L06E	−5.155	−60.642	−6.848	−71.318
ENGA-L08E	−7.156	−81.061	−8.982	−79.097

**Table A7:** Docking scores and Glide e-model in kcal/ mol of molecules ENGA-L06E and ENGA-L08E compared to the co-crystal ligand in proteins AKT-1 and AKT-2.

	AKT1 (pdb ID: 4EJN)		AKT2 (pdb ID: 2X39)	
	Docking Score (kcal mol^−1^)	Glide e-Model (kcal mol^−1^)	Docking Score (kcal mol^−1^)	Glide e-Model (kcal mol^−1^)
Co-crystalized ligand	−12,188	−131,104	−10,158	−103,940
ENGA-L06E	−7.060	−84.653	−4.273	−59.558
ENGA-L08E	−9.446	−86.525	−4.017	−57.739

**Table A8:** Human ovarian cancer cell lines to be used for the in vitro analysis of the compounds biological activity.

Cancer Type	Human Cell Line Designation	Mutated Oncogenes	Special Characteristics
Ovarian Adenocarcinoma	SK-OV-3	TP53 (p.S90fs) PIK3CA (p.H1047R)	Tumor Necrosis Factor; Diphtheria Toxin; Cisplatin and Adriamycin not sensitive; Estrogen receptor α (ERα) positive /Progesterone receptor negative MSI-High *
Epithelial Ovarian Adenocarcinoma	OVCAR-3	MEK1 (p.G159R; p.R160K; p.A158) TP53 (p.R248Q)	Androgen/Estrogen/Progesterone receptor positive; Cisplatin Adriamycin not sensitive; Melphalan resistant; MSS **
Epithelial Ovarian Adenocarcinoma	OVCAR-5	KRAS (p.G12V)	Cisplatin not sensitive; Estrogen receptor α (ERα) negative/GPER positive; MSS **
Ovarian Carcinoma	UWB1.289	p53 (625delAG) BRCA1-null (2594delC)	p53, cytokeratin 7 (CK-7) positive; calretinin positive; Wilms’ tumor protein (WT) positive; Estrogen/Progesterone receptor negative; MSS **
Ovarian Carcinoma	UWB1.289+BRCA1	p53 (625delAG)	Estrogen/Progesterone receptor negative

* MSI-high: High Microsatellite Instability, ** MSS: Microsatellite stable.

**Table A9:** Crystallographic structures of ERK1/2 and AKT1/2 obtained from Brookhaven RCSB PDB (Protein Data Bank).

Protein	PDB ID’s	Year	Resolution (Å)	Co-Crystallized Inhibitor
ERK1	4QTB	2014	1.40	38Z
ERK2	4QTA	2014	1.45	38Z
AKT1	4EJN	2012	2.19	0R4
AKT2	2X39	2010	1.93	X39
