# Supplementary material for: Azasteroid Alkylators as Dual Inhibitors of AKT and ERK Signaling for the Treatment of Ovarian Carcinoma
Source: Cancers (Basel). 2020 May 16;12(5):1263. doi: 10.3390/cancers12051263 (PMC7281072; doi:10.3390/cancers12051263)
Supplement: Supplementary file 1 [file cancers-12-01263-s001.pdf]

cancers MDPI

Supplementary Materials

Azasteroid Alkylators as Dual Inhibitors of AKT and ERK Signaling for the Treatment of Ovarian Carcinoma

Panagiotis Dalezis, Eleni Geromichalou, Aikaterini Polonifi, Sofia Sagredou, Nikolaos Nikoleousakos, Michael Nikolaou, Vasiliki Sarli, Mihalis I. Panayiotidis and Dimitrios T. Trafalis

A

		CONTROL		ENGA-L06E (1h)		ENGA-L06E (3h)		ENGA-L08E (1h)		ENGA-L08E (3h)	
		intensity	ratio	intensity	ratio	intensity	ratio	intensity	ratio	intensity	ratio
OVCAR-3 cells	pAKT	563180	1.21	289472	0.52	152939	0.22	274443	0.40	199215	0.34
	pERK1,2	860724	1.85	414942	0.74	708919	1.02	345929	0.51	164940	0.28
	GAPDH	464712	-	561721	-	691668	-	683957	-	593050	-
SKOV-3 cells	pAKT	617312	0.996	215992	0.40	117254	0.20	118946	0.22	106241	0.20
	pERK1,2	1104413	1.78	530139	0.99	222301	0.39	286115	0.54	257360	0.49
	GAPDH	619567	-	536577	-	576819	-	532376	-	522807	-

Data from western blots representing respective densitometry readings expressed as intensity values and ratios ([pAKT] or [pERK1+pERK2] / [GAPDH] band intensity).

B

		CONTROL		ENGA-L06E		ENGA-L08E	
**OVCAR-3 cells**		Mean intensity	ratio	Mean intensity	ratio	Mean intensity	ratio
30min	pAKT	601325	1.23	430001	0.92	426975	0.93
	pERK1,2	834569	1.71	655377	1.40	460820	1.01
	GAPDH	487948	-	467392	-	456755	-
1h	pAKT	567151	1.14	359454	0.62	405926	0.60
	pERK1,2	819566	1.65	556573	0.96	635950	0.94
	GAPDH	495672	-	579764	-	676543	-
3h	pAKT	539850	1.27	427141	0.61	315402	0.52
	pERK1,2	845927	1.99	777256	1.11	503430	0.83
	GAPDH	425564	-	700231	-	606542	-
6h	pAKT	587921	1.26	228657	0.54	197866	0.47
	pERK1,2	863557	1.85	225372	0.54	317632	0.76
	GAPDH	467213	-	420078	-	418763	-
12h	pAKT	554331	1.18	187663	0.50	185206	0.48
	pERK1,2	854432	1.82	184005	0.49	293415	0.76
	GAPDH	469805	-	378658	-	387541	-
24h	pAKT	570034	1.29	209094	0.59	194218	0.54
	pERK1,2	846679	1.92	207700	0.58	304429	0.84
	GAPDH	442047	-	354679	-	360356	-
		CONTROL		ENGA-L06E		ENGA-L08E	
**SKOV-3 cells**		Mean intensity	ratio	Mean intensity	ratio	Mean intensity	ratio
15min	pAKT	595562	0.98	523773	0.94	546274	1.04
	pERK1,2	1029674	1.70	1064750	1.91	992317	1.89
	GAPDH	605638	-	556732	-	525870	-
30min	pAKT	595562	0.98	435387	0.78	349409	0.66
	pERK1,2	1029674	1.70	969159	1.74	847492	1.61
	GAPDH	605638	-	556732	-	525870	-
1h	pAKT	557009	1.02	400360	0.73	371701	0.63
	pERK1,2	969023	1.78	740686	1.35	636100	1.08
	GAPDH	545033	-	548965	-	587762	-

3h	pAKT	569703	0.99	376873	0.65	287955	0.52
	pERK1,2	996401	1.74	392597	0.68	487004	0.89
	GAPDH	573459	-	578540	-	548799	-
12h	pAKT	584509	0.94	302956	0.58	259479	0.50
	pERK1,2	1002453	1.61	341709	0.65	442756	0.85
	GAPDH	623461	-	524054	-	518875	-

Data from pAKT and pERK1,2 time-depended inhibition curves representing densitometry readings expressed as mean intensity values and ratios ([pAKT] or [pERK1+pERK2] / [GAPDH] band intensity).

Figure 1. Densitometry readings / intensity ratios of expression and quantification data of Figure 3.

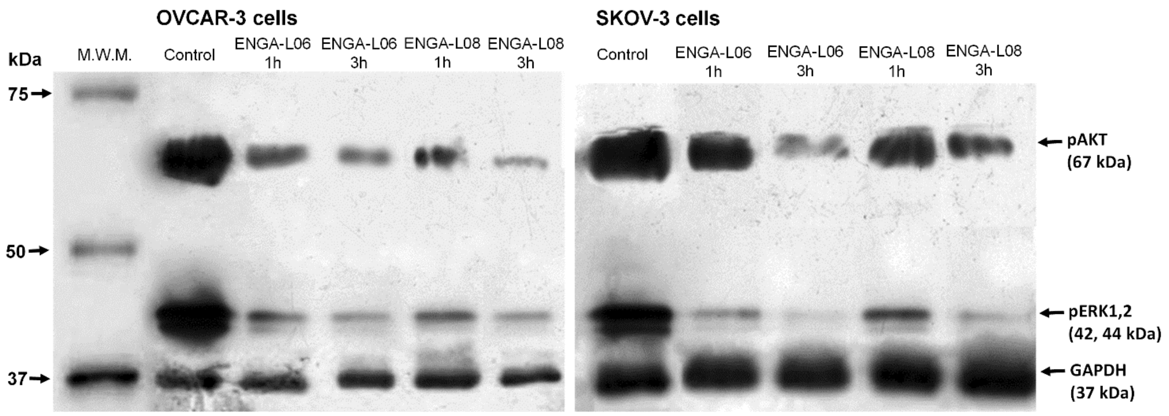

Figure 2. Whole western blots, with molecular weight markers (M.W.M.), presenting the expression levels of pERK 1,2 and pAKT in SK-OV-3 and OVCAR-3 ovarian cancer cell lines after treatment with hybrid aza-steroidal alkylators ENGA-L06E and ENGA-L08E at a concentration of 25 μM in 1 and 3h intervals.
